# Molecular Comparisons of Full Length Metapneumovirus (MPV) Genomes, Including Newly Determined French AMPV-C and –D Isolates, Further Supports Possible Subclassification within the MPV Genus

**DOI:** 10.1371/journal.pone.0102740

**Published:** 2014-07-18

**Authors:** Paul A. Brown, Evelyne Lemaitre, François-Xavier Briand, Céline Courtillon, Olivier Guionie, Chantal Allée, Didier Toquin, Marie-Hélène Bayon-Auboyer, Véronique Jestin, Nicolas Eterradossi

**Affiliations:** French Agency for Food, Environmental and Occupational Health Safety (ANSES), Avian and Rabbit Virology Immunology and Parasitology Unit (VIPAC), Université Européenne de Bretagne, Ploufragan/Plouzané laboratory, Ploufragan, France; University of Maryland, United States of America

## Abstract

Four avian metapneumovirus (AMPV) subgroups (A–D) have been reported previously based on genetic and antigenic differences. However, until now full length sequences of the only known isolates of European subgroup C and subgroup D viruses (duck and turkey origin, respectively) have been unavailable. These full length sequences were determined and compared with other full length AMPV and human metapneumoviruses (HMPV) sequences reported previously, using phylogenetics, comparisons of nucleic and amino acid sequences and study of codon usage bias. Results confirmed that subgroup C viruses were more closely related to HMPV than they were to the other AMPV subgroups in the study. This was consistent with previous findings using partial genome sequences. Closer relationships between AMPV-A, B and D were also evident throughout the majority of results. Three metapneumovirus “clusters” HMPV, AMPV-C and AMPV-A, B and D were further supported by codon bias and phylogenetics. The data presented here together with those of previous studies describing antigenic relationships also between AMPV-A, B and D and between AMPV-C and HMPV may call for a subclassification of metapneumoviruses similar to that used for avian paramyxoviruses, grouping AMPV-A, B and D as type I metapneumoviruses and AMPV-C and HMPV as type II.

## Introduction

The Genus *Metapneumovirus* (MPV), in the family *Paramyxoviridae,* subfamily *Pneumovirinae*, includes globally important viruses in avian and human health. Avian MPV (AMPV) [1]–[5] cause respiratory and genital disorders in poultry having a severe economic impact on the industry [6]. Human MPV (HMPV), is responsible for bronchiolitis in infants [7], [8] and severe infections in the elderly or immunocompromised adults [7], [9]–[11]. AMPV and HMPV are now classified into the genus *Metapneumovirus* [12], to acknowledge the difference in genome order [13] and the absence of the non structural protein genes NS1 and NS2 as compared with members of the *Pneumovirus* genus [14].

MPV have non segmented, single stranded, negative sense RNA genomes between 13.1 and 14.2 kb which are known to encode 9 proteins. MPV genomes are organized in the order 3′-leader-N-P-M-F-M2-SH-G-L-trailer-5′. Genetic and antigenic studies have revealed four AMPV subgroups (A to D) and two HMPV subgroups (A and B) with a high similarity between HMPV subgroup A (strain 001) and AMPV-C [15]–[19]. Genetic sublineages have been defined within HMPV subgroups and AMPV-C, the latter forming two genetic lineages in Muscovy ducks in France [20] and turkeys and wild birds in the USA [21]–[25]. It is not fully understood why AMPV-C pathogenic for turkeys emerged in the USA, whereas such viruses have not been recognized in the EU or Asia, with the exception of the AMPV-C strain recently isolated in chickens in China [26].

Determining full length sequences of viral genomes is an essential step towards studying the possible molecular basis for host tropism or pathogenicity, first by allowing the development of reverse genetics systems for the studied strains, and second by allowing genome wide sequence comparisons highlighting relevant regions to study using reverse genetics. Full length genome sequences of subgroup A and B viruses are available [27]–[30]. Full sequences are available for AMPV-C from both turkey [16], [31] and wild goose [21], [32] in the US, from pheasants in Korea [33], and most recently from Muscovy duck in China (Acc N° KC915036 and KF364615). Partial sequence (M gene) is available for a Chinese chicken isolate of AMPV-C (acc N° JX422020).

In combination with sequences described previously [20], [24], [34], [35], this study completes the sequences of European AMPV-C (French duck isolate, Fr-AMPV-C) and D (French Turkey isolate, Fr-AMPV-D). Comparisons were made to full length sequences of European AMPV-A, B and US-C and of all HMPV-A and B sublineages.

## Materials and Methods

### Acquisition of Full length Fr-AMPV-C and D sequences

Fr-AMPV-C Muscovy duck/France/1999/99178 and Fr-AMPV-D Turkey/France/1985/85035 (the latter previously identified Fr/85/1 in refs [35], [36]) were propagated in Vero cells as described previously [20], [36]. The 99178 and 85035 viruses were shown experimentally to be pathogenic for SPF Muscovy ducklings or turkeys, respectively [37]. Fr-AMPV-C and Fr-AMPV-D virus stocks had a titer of log_10_^4.20^ and log_10_^5.00^ TCID_50_/ml, respectively [38]. Viral RNA was extracted using QIAamp Viral RNA mini kit (Qiagen, France) according to the manufacturer’s instructions. Primers were designed from previously published partial sequences (Table S1) and from the full genome sequences of US AMPV-C and AMPV-A and B. Additional primers were defined from the newly determined sequences (sequence of all primers are available on request). Sequence of the leader and trailer as previously reported, based on 3′ tailing of the genome and its positive replication intermediate [34]. cDNA copies of the viral RNA were prepared using superscript II (Invitrogen, France) according to manufacturer’s recommendations. dsDNA was amplified from cDNA in overlapping segments using Expand high fidelity enzyme (EHF, Roche, France) according to manufacturer’s recommendations. PCR products were purified using an Utraclean Gelspin kit (Mobio), and sequenced using Big Dye Terminator v3.1 cycle sequencing kit as recommended by the manufacturer. Each genome region was amplified three times and PCR products were sequenced in both directions.

### Genetic comparisons

Full length Fr-AMPV-C and Fr-AMPV-D sequences were assembled using vectorNTIv11 software, then aligned using MEGA 5.2 [39] against available full genome sequences of MPV downloaded from Genbank (four HMPV and 13 AMPV genomes, see Acc No in Table 1). Open reading frames (ORFs) were predicted and then compared with those reported previously using MEGA 5.2. Program “getorf” from EMBOSS (emboss.sourceforge.net) was used to detect potential ORFs which had been defined as a region of at least 150 nucleotides between two STOP codons. The amino acid (aa) sequences of these ORFs were compiled in a database file. ORFs identified from Fr-AMPV-C and Fr-AMPV-D were compared to all other MPV ORFs by local BLAST [40] and were submitted to global BLAST search online.

**Table 1:** Molecular features of MPV regions and the deduced gene products.

			Origin											M2									
Virus	Strain	Accessionnumber	Country	Hostspecies	3′	N^a^	N/P^b^	P	P/M	M	M/F	F	F/M2	M2-1	M2-2	M2-2/SH	SH	SH/G	G	G/L	L	5′	Genome^c^
Fr-AMPV-C	99178	HG934338	France	MuscovyDuck	54	1185(394)	22	885 (294)	25	765(254)	105	1614(537)	33	555(184)	216 (71)	21	528(175)	187	1758(585)	44	6018(2005)	181	**14152**
AMPV-C	GYP	KC915036	China	MuscovyDuck	53	1185(394)	22	885 (294)	27	765(254)	105	1614(537)	33	555(184)	216 (71)	22	528(175)	187	1758(585)	44	6018(2005)	>141	**>14114** d
AMPV-C	S-O1	KF364615	China	MuscovyDuck	>13	1185(394)	22	885 (294)	27	765(254)	105	1614(537)	32	555(184)	216 (71)	22	528(175)	186	1758(585)	44	6018(2005)	>148	**>14079**
AMPV-C	APV/11	DQ009484	USA(Minnesota)	Goose	>13	1185(394)	22	885 (294)	25	765(254)	105	1614(537)	33	555(184)	216 (71)	23	528(175)	185	1758(585)	44	6018(2005)	>140	**>14070**
AMPV-C	15a/01	NC-007652	USA(Minnesota)	Goose	>13	1185(394)	22	885 (294)	25	765(254)	105	1614(537)	33	555(184)	216 (71)	23	528(175)	185	1758(585)	44	6018(2005)	>140	**>14070**
AMPV-C	APV-CO	AY590688	USA(Colorado)	Turkey	53	1185(394)	22	885 (294)	25	765(254)	105	1614(537)	32	555(184)	216 (71)	23	528(175)	186	1758(585)	44	6018(2005)	180	**14150**
AMPV-C	APV/CO	AY579780	USA(Colorado)	Turkey	53	1185(394)	22	885 (294)	26	765(254)	105	1614(537)	33	555(184)	216 (71)	22	528(175)	186	756(251)	44	6018(2005)	180	**13134**
AMPV-C	2a/97	FJ977568	USA(Minnesota)	Turkey	53	1185(394)	22	885 (294)	25	765(254)	105	1614(537)	32	555(184)	216 (71)	22	528(175)	186	1758(585)	28	6018(2005)	180	**14151**
AMPV-C	PL-1	EF199771	Korea	Pheasant	53	1185(394)	22	885 (294)	26	765(254)	105	1614(537)	33	555(184)	216 (71)	22	528(175)	184	795(264)	28	6018(2005)	180	**13170**
AMPV-C	PL-2	EF199772	Korea	Pheasant	53	1185(394)	22	885 (294)	26	765(254)	105	1614(537)	33	555(184)	216 (71)	22	528(175)	184	795(264)	28	6018(2005)	180	**13170**
AMPV-A	8544	DQ666911	United Kingdom	Turkey	55	1176(391)	24	837 (278)	25	765(254)	60	1617(538)	26	561(186)	222 (73)	52	528(175)	76	1176(391)	85	6015(2004)	115	**13371**
AMPV-A	LAH	AY640317	UnitedKingdom	Turkey	55	1176(391)	25	837 (278)	25	765(254)	60	1617(538)	26	561(186)	222 (73)	52	528(175)	76	1176(391)	86	6015(2004)	115	**13373**
AMPV-A	259-01/03	JF424833	Italy	Turkey	55	1176(391)	24	837 (278)	25	765(254)	60	1617(538)	26	561(186)	222 (73)	52	528(175)	76	1176(391)	85	6015(2004)	115	**13371**
AMPV-B	VCO3/60616	AB548428	France	Turkey	55	1176(391)	23	840 (279)	25	765(254)	61	1617(538)	27	561(186)	222 (73)	37	543(180)	105	1245(414)	100	6015(2004)	135	**13508**
Fr-AMPV-D	85035	HG934339	France	Turkey	62	1176(391)	24	837 (278)	25	765(254)	54	1617(538)	26	567(188)	222 (73)	57	528(175)	86	1170(389)	106	6021(2006)	122	**13415**
HMPV A1	00-1	AF371337	Netherlands	Human	39	1185(394)	23	885 (294)	32	765(254)	122	1620(539)	65	564(187)	222 (73)	30	552(183)	201	711(236)	209	6018(2005)	>166	**>13350**
HMPV-A2	NL/00/17	FJ168779	Netherlands	Human	54	1185(394)	23	885 (294)	31	765(254)	122	1620(539)	37	564(187)	222 (73)	32	540(179)	211	660(219)	241	6018(2005)	185	**13336**
HMPV-B1	NL/1/99	AY525843	Netherlands	Human	53	1185(394)	23	885 (294)	32	765(254)	121	1620(539)	26	564(187)	222 (73)	32	534(177)	206	675(224)	239	6018(2005)	152	**13293**
HMPV-B2	NL/94/01	FJ168778	Netherlands	Human	53	1185(394)	23	885 (294)	32	765(254)	121	1620(539)	26	564(187)	222 (73)	30	534(177)	210	711(236)	188	6018(2005)	154	**13282**

a For all coding regions, in addition to the ORF length, the deduced protein length was indicated in brackets.

b number of nucleotides between the N and P ORFs, this encompasses the 3′NCR of the N gene, the N-P intergenic region and the P 5′NCR.

c Due to the overlap between M2.1 and M2.2, the sum of nucleotides presented is greater than the genome length.

d > Indicates the full genome is longer than shown due to the genome extremities not being confirmed.

3′ and 5′ including the leader and trailer sequences respectively.

### Codon usage

The extent of codon bias was evaluated among the studied MPV. To measure the general non-uniformity of the synonymous codon usage, the effective number of codons (Nc) [41] was calculated based on the longest MPV gene (L). Nc values range from 20 when only one of the possible synonymous codons is used for each amino acid, to 61 when all synonymous codons are used equally. The closer the Nc value is to 20, the stronger the bias in codon usage and the more non random codon usage is. It is generally admitted that genes have a significant codon bias when the Nc value is less than or equal to 35 [42].

In the Nc value calculation formula: *Nc* = 2+9/*F*2+1/*F*3+5/*F*4+3/*F*6, F2 corresponds to the probability that two randomly chosen codons for an amino acid, possibly encoded by two distinct codons, are identical. F3 is the probability that three randomly chosen codons for an amino acid with three synonymous codons are identical and so on for F4 and F6. The Nc value was determined using CodonW 1.4.4 (http://codonw.souceforge.net) and was correlated to the percentage of G+C at the third position (GC3) as it has been shown previously to be a major factor influencing the synonymous codon usage pattern in the HMPV genome [43].

#### Phylogenetics

All available AMPV full-length genome sequences and one representative of each of the four HMPV sublineages were aligned using Clustal W. Alignments were also checked manually for a good correspondence of the common coding regions. Phylogenetic analysis was performed using MEGA 5.2 with the Neighbor-Joining method (1000 boostrap replicates) and the Kimura-2-parameter substitution model.

## Results and Discussion

### Sequence overview

The full length consensus sequences for Fr-AMPV-C and Fr-AMPV-D were 14152bp- and 13415bp-long, respectively. Table S1 presents the previously released sequences for these two viruses. The present report provides newly determined sequences equal to 73 and 78% of the total genome sequence for these viruses, respectively. The full length genomes were consistent in the order (3′-leader-N-P-M-F-M2-SH-G-L-trailer-5′) and in the size of known ORFs for MPV genomes (Table 1). Both sequences have been submitted to EMBL (Accession numbers HG934338 and HG934339 respectively). The Fr-AMPV-C and Fr-AMPV-D genomes, like several other AMPVs, were found not to conform to the “rule of six” [44], a feature that separates pneumovirinae from paramyxovirinae [45]. In general, genome lengths were conserved amongst AMPV subgroups A, B and D and amongst HMPV sublineages, however clear differences could be seen in the genome lengths of AMPV subgroup C viruses, mostly resulting from the different lengths of their G genes (Table 1).

### Phylogenetics

**Figure 1:**
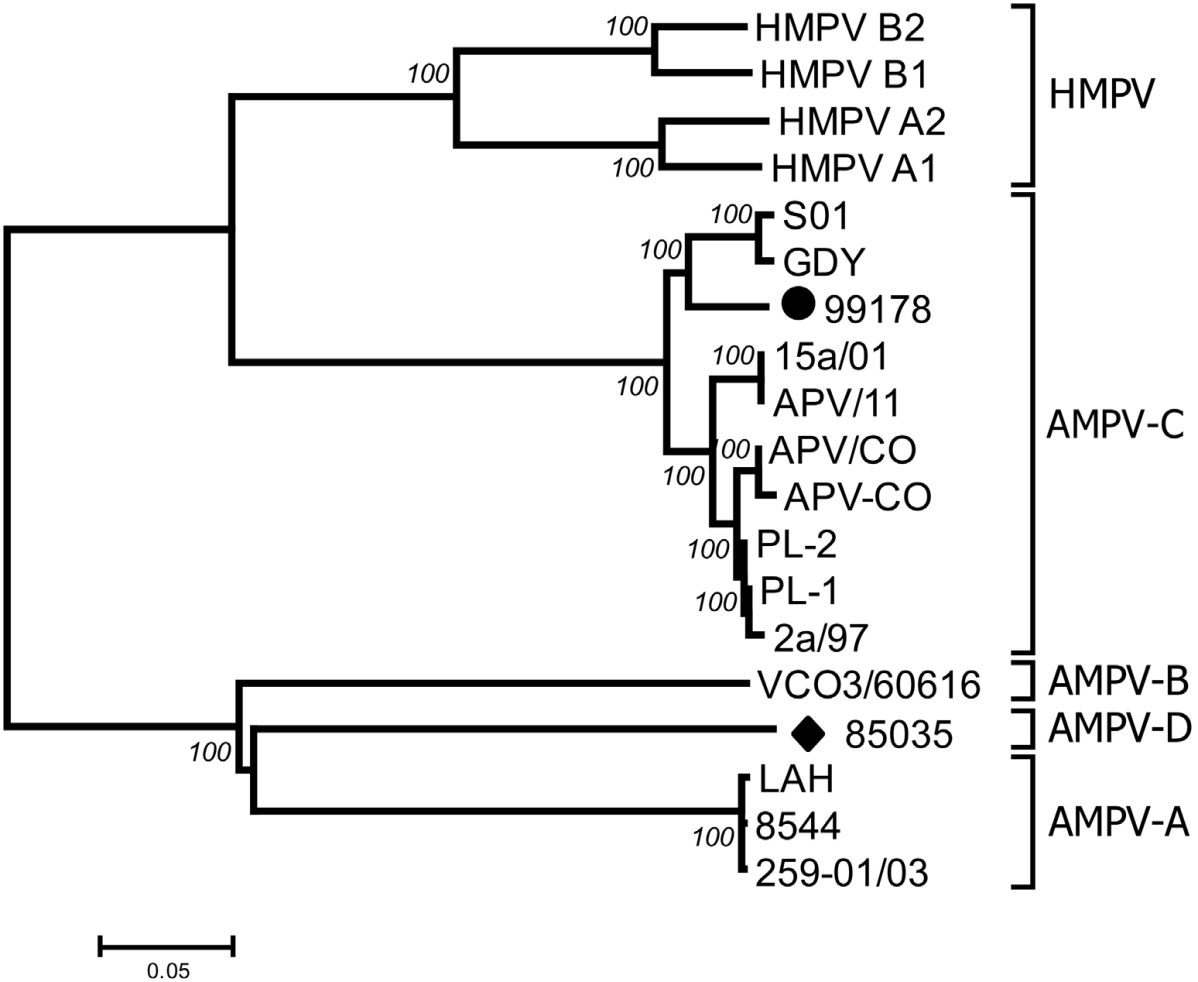
Genetic relationships between previously published MPV genome sequences and the full length sequences of Fr-AMPV-C (•) and D (♦). The tree was constructed as described in the text using the neighbor-joining method. Percentages at branch points represent the number of times the group to the right of that branch point occurred among 1000 trees generated by bootstrap from the original alignment.

Three significant clusters were observed, one grouping all HMPVs, a second grouping AMPV-Cs and a third grouping the AMPV-A, B and D subgroups (Fig. 1).

Within the AMPV-C cluster, viruses isolated from Muscovy ducks (SO1, GDY and 99178) formed a separate sub-lineage from the others. This separation is potentially related to species rather than geographic origin as the Asian SO1, GDY, PL-1 and PL-2 isolates were split into different clusters (SO1 and GDY with the European 99178 isolate and PL-1 and 2 with the US isolates, Fig. 1), although these geographical relationships could be blurred if AMPV were shed by migratory birds, in the overlap between the East Asian-Australasian flyway with both the East Atlantic and/Pacific Americas flyway in the Northern hemisphere [46].

### Nucleoprotein (N) ORF

The first ORF in the AMPV genome encodes the N protein, which is a component of the polymerase complex and important for the formation of the nucleocapsid helical structure [45], [47]. The N ORF of Fr-AMPV-C was more closely related to that of other AMPV-Cs and HMPVs in terms of length and aa conservation than it was to AMPV-A, B or D.

**Figure 2:**
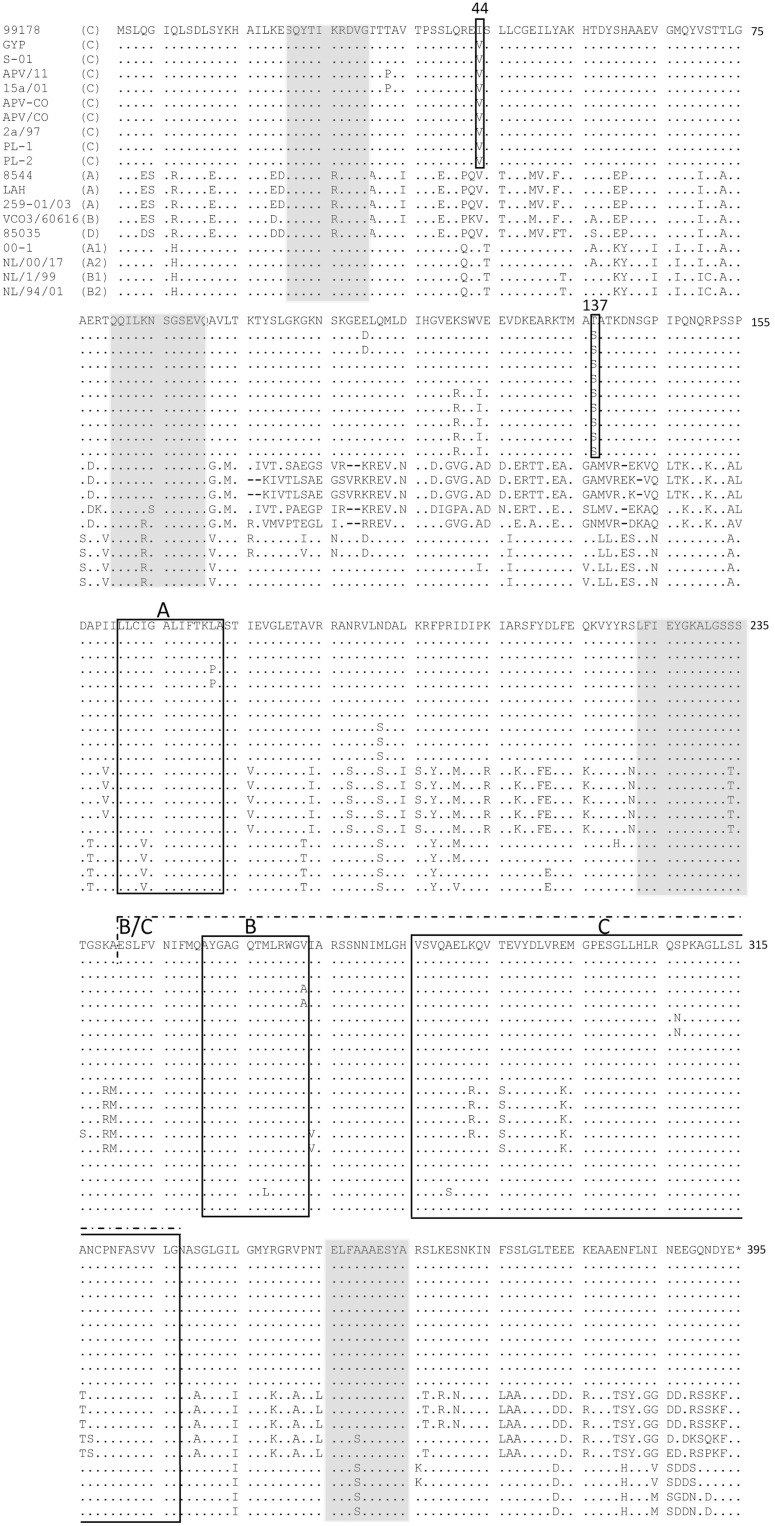
Amino acid comparisons of the nucleocapsid ORF of AMPV-A, B, C and D and HMPV subgroups A and B. Positions 44 and 137 were specific to Fr-AMPV-C. Grey shaded boxes represent four highly conserved regions across all MPVs. Boxes A, B and C are regions that have been reported to be conserved amongst pneumoviruses (Barr et al 1991). In metapneumoviruses domains B and C appear to be extended creating one single larger domain (B/C). *end of protein.

Fr-AMPV-C, N ORF (394aa) was identical to previously reported AMPV-C N ORFs regardless of host species and to those of all HMPV sublineages (Table 1). High aa identity was observed with all other AMPV-C sequences (99%) and with all HMPV sublineages (89–90%) however, the identities with AMPV-A, B and D were lower (70, 71 and 73%, respectively). Two aa positions (44 and 137) were found to be specific of Fr-AMPV-C N compared to all other AMPV-Cs, notably these were the same amino acids as found in HMPV subgroups at this position (Fig. 2). Neither of these amino acids was within the three conserved regions identified previously among pneumoviruses (Barr et al 1991) and represented as boxes A–C in Fig. 2 the two latter (B and C) being merged in MPV forming a larger conserved domain B/C (aa 241–327 Fig. 2). Four separate regions (see grey shaded boxes in Fig. 2) were also highly conserved in all MPVs.

Subgroups A, B and D viruses were more closely related in terms of length and aa identity than they were to AMPV-Cs or HMPVs. Indeed, the length of the Fr-AMPV-D N protein (391aa) was identical to that of both AMPV-A and B and three amino acids shorter than AMPV-C and HMPV N proteins. Amino acid identities were high with A and B (89–90%) but lower with subgroup C and HMPV N proteins (71–74%). Localization of aa differences can be seen in Fig. 2, which also supports a relationship between Fr-AMPV-D and subgroups A and B.

### Phosphoprotein (P) ORF

The second main ORF in the AMPV genome encodes the P protein, which is also part of the polymerase complex. Consistent with N protein comparisons, the P ORFs of AMPV-Cs and HMPVs were more closely related than they were to AMPV-A, B and D. In the same respect, subgroups A, B and D also demonstrated closer relationships.

The length of the Fr-AMPV-C, P ORF (294aa) was identical to other previously reported subgroup C P ORFs regardless of host species, and to those of all HMPV subgroups (Table 1). The P ORFs of AMPV-A, B and D were 16–17aa shorter (Table 1). The full length Fr-AMPV-C P sequence demonstrated a high aa conservation of 96–97% with all AMPV-C sequences, 67–68% with HMPV subgroups and 56%, 54% and 53% with AMPV-A, B and D respectively. Sequence conservation in the carboxy terminal half of the P protein (aa160–294) was notably higher for all the studied MPVs than it was in the amino terminal half (aa11–159). The carboxy terminal half has been reported to support most of the interactions with the N protein and polymerase complex, as reviewed by Easton et al., 2004 [45]. The high conservation of the P interaction domain between AMPV-C and HMPV is consistent with the finding that a recombinant chimeric HMPV with the P gene derived from AMPV-C was able to replicate in Vero cells [48], [49].

**Figure 3:**
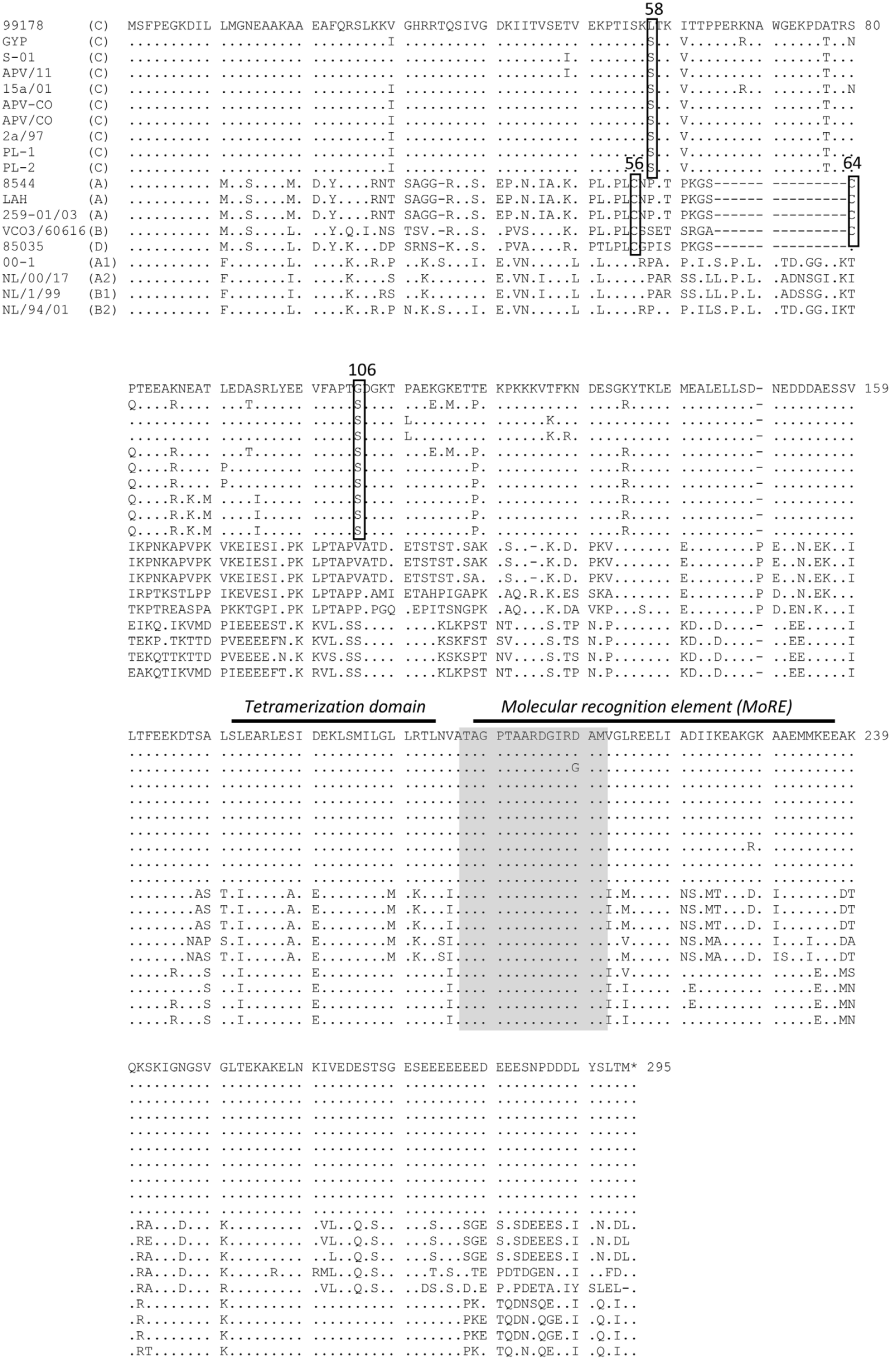
Amino acid comparisons of the phospoprotein ORF of AMPV-A, B, C and D and HMPV subgroups A and B. A region resposible for the tetramerization of the P protein is shown together with a molecular recognition element (MoRE). Grey box represents a highly conserved region in MPVs in the MoRE. *end of protein.

In common with all subgroup C and HMPV P protein sequences analyzed previously [50], Fr-AMPV-C P lacked cysteine residues and maintained high conservation within the region (aa185–240) proposed to play a role in maintaining the structural integrity of the nucleocapsid complex [51]. More recently, this region in the HMPV P sequence has been shown to contain a short molecular recognition element (aa198–211) and a small domain (aa171–193) responsible for P tetramerization [52] (Fig. 3). In the later domain subgroup C viruses were fully conserved and only differed at two amino acid positions from all HMPV sequences. Sequences of subgroups A, B and D in this domain were fully conserved but they differed at three and five amino acid positions from HMPVs and AMPV-Cs respectively (Fig. 3). Interestingly the first 14aa of the molecular recognition element were 100% conserved in all MPVs with the exception of strain SO1 which contained just one aa difference (Fig. 3 grey shaded box).

The length of the Fr-AMPV-D P protein (278aa) was identical to subgroup A and just one aa shorter than the P protein of subgroup B. Amino acid identities were 71–72% conserved with subgroups A and B. In contrast to subgroup C and HMPV P proteins, subgroups A, B and D contained cysteine residues (Fig. 3). Cysteine 56 was conserved in subgroups A, B and D and cysteine 64 was conserved between subgroups A and B. Subgroups A, B and D all differed extensively at the extreme C terminus of the P protein (Fig. 3).

### Matrix protein (M) ORF

The third main ORF in the AMPV genome encodes the M protein which orchestrates the assembly of viral components at the plasma membrane, through interactions with the viral glycoproteins and nucleocapsid [53], [54]. The length of the M ORF (254aa) was identical in all MPVs and its aa sequence was extremely conserved amongst all AMPV-Cs (99%) and between AMPV-A, B and D (90 to 94%). High aa conservation was also seen between AMPV-C and HMPVs (87–88%), but to a lesser extent between AMPV-C and AMPV-A, B and D (78–79%). The hexapeptide (aa14–19) with no known function but conserved across all pneumoviruses [55] was also highly conserved in all MPVs, with the exception of Fr-AMPV-C that contained one conservative aa change (V→I) at position 18. Three cysteine residues (aa110, 147 and 239) were also conserved in all MPVs.

### Fusion protein (F) ORF

The fourth main ORF in the AMPV genome encodes the highly antigenic, type I membrane fusion protein F. In paramyxoviruses, F is synthesized as an inactive single precursor F0, which is directed to the endoplasmic reticulum by its N-terminal signal peptide. F0 is then cleaved at an arginine-rich cleavage site, mostly by host endoproteases such as furins, into functional F1–F2 subunits held together by disulfide bonds. The F1 subunit remains inserted into the virus membrane by its carboxy-terminal transmembrane domain [56]. The F2 subunit of both HMPV and human and bovine RSV (HRSV, BRSV) has been reported to determine cellular host range [57], [58].

**Figure 4:**
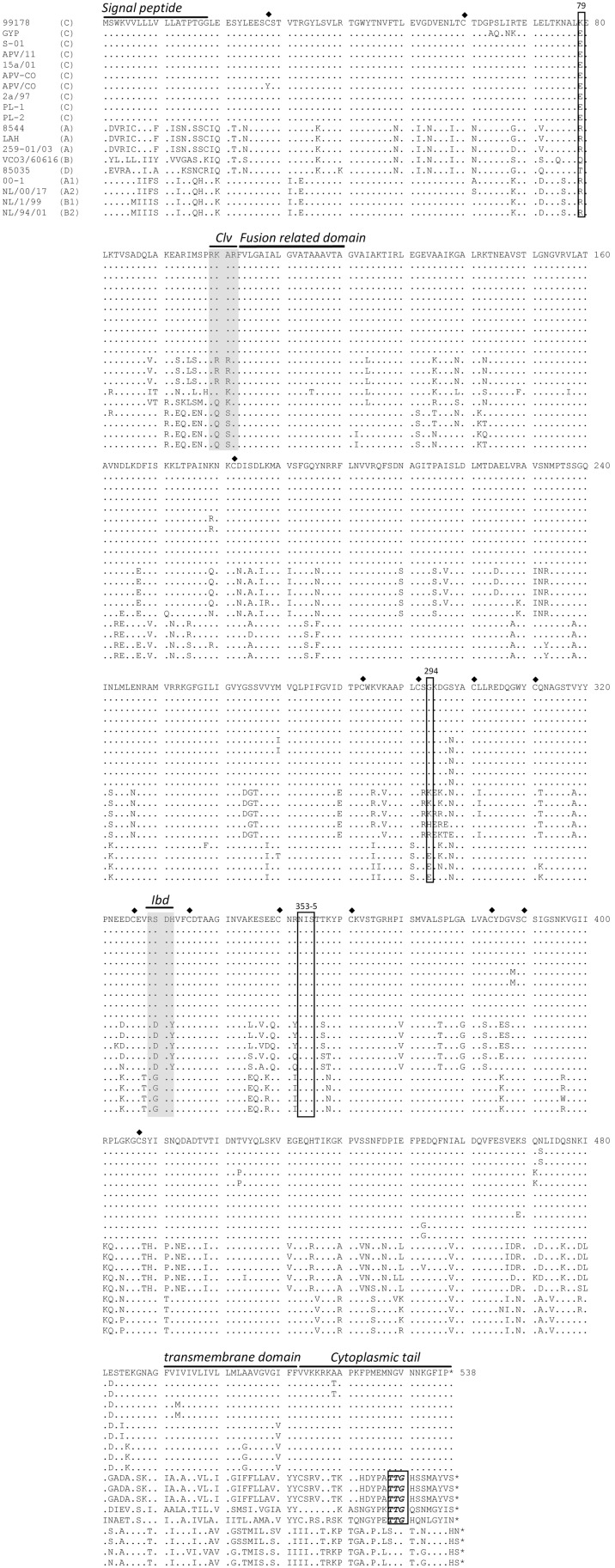
Amino acid comparisons of the fusion ORF of AMPV-A, B, C and D and HMPV subgroups A and B. Previously identified biologicaly important domains are labled and underlined. Diamonds indicate conserved cysteine residues. Open boxes highlight other domains discused in the relevent paragraph. Cleavage site (Clv) and integrin binding domain (Ibd). *end of protein.

The length of the Fr-AMPV-C, F ORF (537aa) was identical to previously reported AMPV-C F ORFs, one aa shorter than the F of AMPV-A, B and D, and two aa shorter than HMPV F (Table 1). The aa sequence of Fr-AMPV-C was extremely conserved (98–99%) with all other AMPV-Cs, highly conserved (81–82%) with all HMPV sublineages and slightly less conserved (71–73%) with AMPV-A, B and D. The cleavage site was located in the same position (aa 99–102) in all MPVs (grey box *Clv* in Fig. 4). The cleavage sequence (RKAR) conserved in all AMPV-Cs was not consistent with the typical furin cleavage (R-X-R/K-R) site found in AMPV-A, B and D (RRRR, RKKR and RQKR respectively), however a less typical recognition site (R-X-X-R) has also been shown to be functional [59].

The sequence of the signal peptide (aa 1–18 Fig. 4) at the N terminal end of F2 was extremely subgroup specific in the avian viruses, a rather surprising finding considering its function, with at best 39% identity between subgroups D and C however, a higher identity (56–61%) was seen between subgroup C and HMPV. Two cysteine residues (aa 28 and 60) (Fig. 4) remained conserved in all MPVs, including Fr-AMPV-C and D (with the exception of position 28 in the AY579780 APV/CO sequence), which further supports their already suggested possible structural role [50].

In the F1 subunit, the fusion related domain (103–125) [60] was 100% conserved in all MPVs, with the exception of one aa change in AMPV-B. Other interesting conserved features in all MPV sequences included i) the position of the 12 extracellular cysteine residues (Fig. 4), a finding that is consistent with their possible involvement in protein secondary structure through the formation of disulphide bonds (Van den Hoogen et al., 2002), and ii) a proposed N-Linked glycosylation site (aa353–355, Fig. 4) [50]. Other features appeared subgroup or strain specific. For example, all AMPV-C F1 sequences contained a glycine residue (G) at amino acid 294 (Fig. 4), a position previously reported in HMPV to be influential in low pH-triggered fusion and syncitial phenotype [61], [62], and in AMPV-A to contribute to the increased protective capacity of a genetically modified virus [63]. An integrin binding domain ^329^RGD^331^ (grey box *Ibd* in Fig. 4) has been identified in the F1 subdomain of the HMPV F protein, and changes to either of its first two residues have been shown to be detrimental for fusion activity [64]. No such typical RGD domain exists in the AMPV Fprotein: in contrast, all subgroup C sequences contained a motif ^329^RSD^331^ and subgroups A, B and D contained a motif ^329^RDD^331^. The subgroup specific modifications in this biologically significant domain also support the closer relationship between subgroups A, B and D.

The F1 cytoplasmic tail also exhibited inter subgroup variation (Fig. 4). Indeed, intra subgroup identities in this part of F1 were extremely high (subgroup C sequences 96–100%, subgroup A 100%, and HMPV 88–100%), whereas an extremely low conservation between subgroups was observed, with at best 56% between AMPV-B and D and as low as 0–4% between AMPV-A and HMPV. In spite of this low conservation, a TTG motif was conserved in the F1 cytoplasmic tails of AMPV-A, B and D (Fig. 4). The cytoplasmic tails of several paramyxovirus fusion proteins have been shown to be important in virus assembly [65].

Finally, several regions in the F1 subunits of pneumoviruses and MPVs are important in the production of neutralizing antibodies [66]–[69]. Brown et al 2009 [66] demonstrated that two regions (211–310 and 336–479) of the AMPV-A F protein were recognized by neutralizing antibodies to both subgroup A and B but not subgroup C virus. These regions appeared highly conserved (mean 95%) between AMPV-A, B and D but much less so with AMPV-C and HMPV (71–84%). Such identities are consistent with the lack of cross neutralization of AMPV-A, B and D with subgroup C viruses, and further suggest that neutralizing epitopes within regions 211–310 or 336–479 of AMPV-A and B are also likely to exist in subgroup D. These genetic data thus correlate with the previously reported antigenic cross-reactivity between AMPV subgroups [66], [70].

### The M2 protein ORF

The M2 gene contains two overlapping ORFs (M2.1 and M2.2). M2.1 is involved in virus synthesis and enhances the processivity of the viral polymerase whilst M2.2 has been suggested to alter the balance between transcription and replication [45]. M2.2 has also been shown to be important for adaptation to Vero cells [71]. Fr-AMPV-C M2.1 was identical in length (184aa) to all other subgroup C sequences, however two, four and three aa shorter than AMPV-A and B, AMPV-D and HMPV sequences, respectively. Fr-AMPV-C amino acid identities were again highly conserved with all other subgroup C sequences (98%) and with all HMPV sublineages (84–85%), which is consistent with the finding that the polymerase complex proteins (M2.1, N, P and L) of either virus are biologically active in heterologous rescues [49], [72]. Similarly, high identities (87–90%) were also observed between AMPV-A, B and D, however identities between subgroup C with AMPV-A, B and D (71–74%) were moderately lower. The three cysteine residues found in all pneumoviruses [50] within the first 30 aa of M2.1 remained conserved in both Fr-AMPV-C and D. M2.1 is intra cellular and conservation of cysteines has been shown in RSV to be important for the formation of structural metal binding motifs [73], [74].

In M2.2, a similar level of conservation was seen between subgroup C sequences (93–99%) but conservation was notably lower with the HMPV sublineages (54–58%) and with AMPV-A, B and D (20–24%) which was consistent with previous literature [50]. The highest inter subgroup identity was seen between AMPV-A, B and D (64–72%).

Three cysteine residues were conserved across all MPVs (aa7, 16 and 56) and a further two conserved between AMPV-A, B and D (aa22 and 59). Cysteines 7, 16 and 22 fell within a region (aa0–25) identified in HMPV as critical to promote viral gene transcription [75].

### Small hydrophobic protein ORF (SH)

SH is a small type II membrane glycoprotein protein localized in the endoplasmic reticulum, golgi and cell surface [76]. SH has been shown to be non-essential for virus attachment, infectivity or virion assembly [29], [77], [78]. However, a SH deletion in AMPV-A contributed to an altered syncytial phenotype and a reduced immunogenicity [79].

SH length (175aa) was conserved across all AMPVs with the exception of subgroup B (180aa). This was in contrast to the varying lengths seen in HMPV SH (177–183aa). A range of 83–100% aa identity was seen between all subgroup C SH sequences. SH conservation between AMPV-A, B and D was considerably lower (42–49% aa identity), and even lower between HMPVs and all APMVs (14–31%). In the SH transmembrane domain, subgroup-C sequences demonstrated a closer relationship with HMPVs (39–50%) than they did with subgroups A, B or D (19–30%). Subgroups A, B and D were more closely related in their transmembrane domains (70–86%).

Further relationships between AMPV-C/HMPV or AMPV-A/B/D were evident in the conservation of cysteine residues. AMPV-A, B and D had fourteen (3 in the intracellular and 11 in the extra cellular domain) and AMPV-Cs and HMPVs had nine in the extracellular domain. Seven cysteines were conserved across all MPVs in the extracellular domain.

These features make the SH protein the second most variable protein (after the G gene) in the MPV genome, with respect to inter-subgroup aa identity. Interestingly inter-subgroup differences did not prevent the restoration of a typical phenotype when SH_B_ was introduced into a SH-deleted AMPV-A genome background. A similar result could not be achieved using SH_C_ [79].

### Glycoprotein ORF (G)

G is a heavily glycosylated type II membrane protein, involved in, but not essential for virus attachment [29], [80], [81]. Most recently it is emerging as an inhibitor of the cellular host immune response to viral infection [82]–[84].

We have reported previously genetic analysis of the large G ORF in Fr-AMPV-C [24] and D [24], [35]. Both studies showed that G exhibited the most extensive divergence between subgroups in terms of length and sequence identity. Differences in the length of the G protein ectodomain amongst AMPV-C isolates have been also reported [18], [85]–[87]. In the present study, the length of both Chinese AMPV-C G sequences were identical to that of Fr-AMPV-C (585aa), whilst both Korean AMPV-C G sequences were shorter (264aa) and more closely resembled AMPV/CO (table 1). Intra subgroup C identities including the two Chinese and two Korean AMPV-C G sequences were within the range reported previously (75–83%) [24]. The two pairs of Asian AMPV-C sequences were highly conserved (intra pair identity = 97 and 99.6%, respectively) and the four viruses exhibited the conserved intracellular and trans membrane domains and the ten extracellular cysteine residues previously reported to be conserved in all AMPV-Cs [24]). Remarkably, 19 out of 22 aa differences between the two Chinese sequences were confined to a short domain (aa269–299) immediately at the N terminal end of the previously identified, variable part of G ectodomain.

### The polymerase protein ORF (L)

The final ORF of metapneumovirus genomes encode the large RNA-dependent RNA polymerase protein L. It is a major part of the polymerase complex responsible for most of the enzymatic processes involved in transcription and replication [88]. It is also responsible for viral messenger RNA capping, polyadenylation, methylation and phosphorylation processes [89].

The length of Fr-AMPV-C L (2005aa) was identical to all other AMPV-C and HMPV sequences, one aa shorter than that of AMPV-A and B and two shorter than that of Fr-AMPV-D. Extremely high aa conservation was observed amongst subgroup-C viruses (98–100%), and amongst HMPV sublineages (94–99%). Closer relationships were observed between subgroup-C viruses and HMPVs (80–81%) than between these viruses and subgroups A, B, D (63–64%). Subgroup D again demonstrated a closer relationship with subgroups A and B (84–86%).

**Figure 5:**
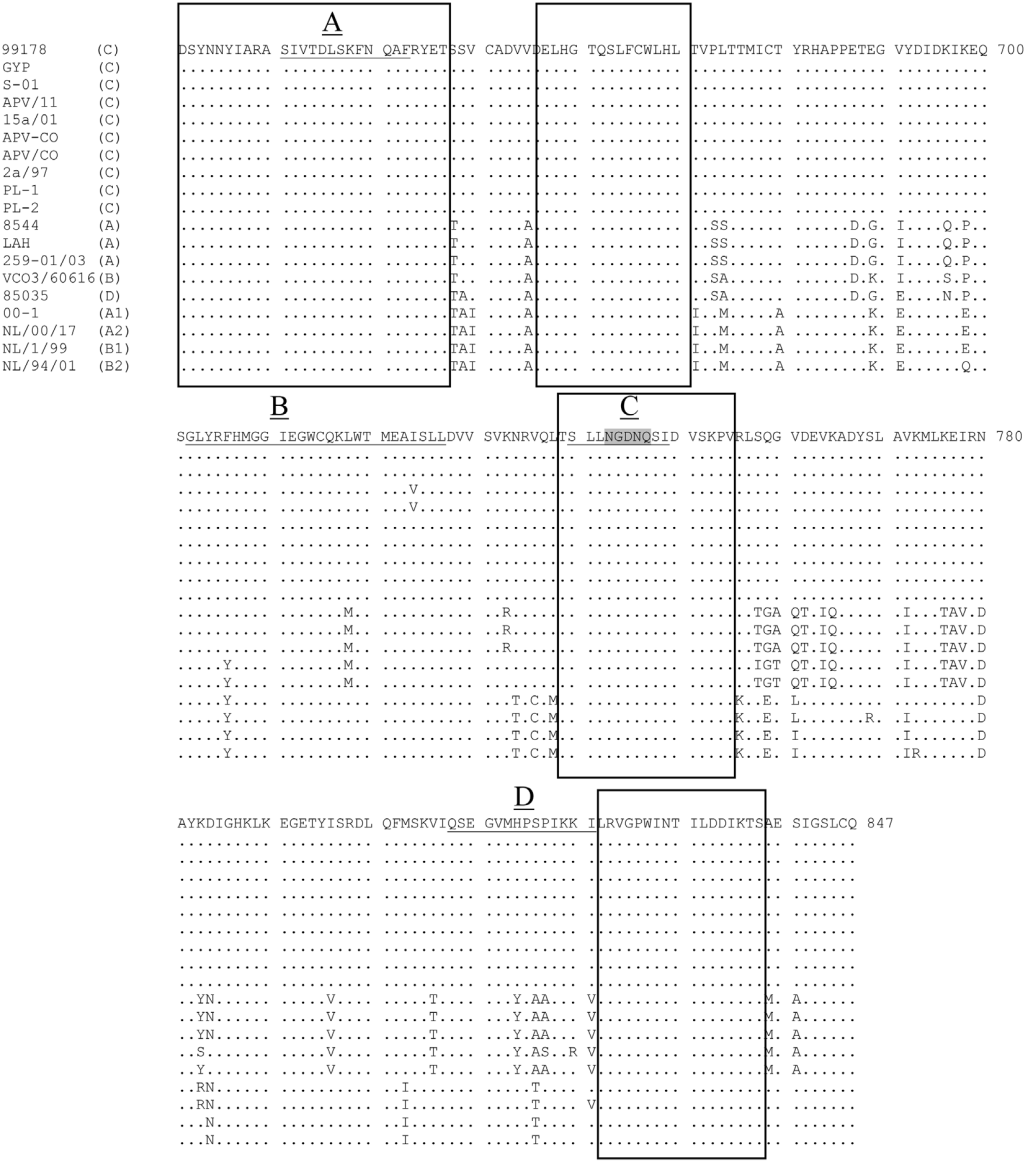
Amino acid comparisons of the previously reported conserved domain III (Poch et al., 1990) in the L ORF of AMPV-A, B, C and D and HMPV subgroups A and B. Four core motifs (A, B, C and D) described by Poch et al 1990 are underlined. A pentapeptide conserved in mononegavirales is highlighted in grey within motif C. Open boxes highlight regions in MPVs with 100% conservation. *end of protein.

Six functional domains (I–VI) have been identified in the L proteins of non segmented negative strand viruses [88], with domain III including four highly conserved core polymerase motifs (A–D) [50] The newly identified sequences were consistent with these findings in motifs A, B and C, however some variation was seen in motif D (Fig. 5) and motifs A and C appeared to be larger in MPVs (Fig. 5). Two additional regions were observed where all MPVs were completely conserved (Fig. 5). The QGDNQ pentapeptide found in motif C within domain III was replaced in all MPVs by NGDNQ. AMPV-A, B and D shared four or five amino acids in motif D that were not represented in the subgroup C viruses or HMPVs (Fig. 5). Further conservation was observed between all MPVs in the ATP-binding motif (aa 1677–1721) identified previously [50] and in five previously unidentified regions scattered through the L ORF were all MPVs were 100% conserved over 15 or more aa (aa15–29, aa549–573, aa656–670, aa1250–1265 and aa1297–1319). Finally two regions of subgroup specific sequences were observed towards the N terminal end of the L protein (302–320, and 431–446).

### Codon usage in the L gene

**Figure 6:**
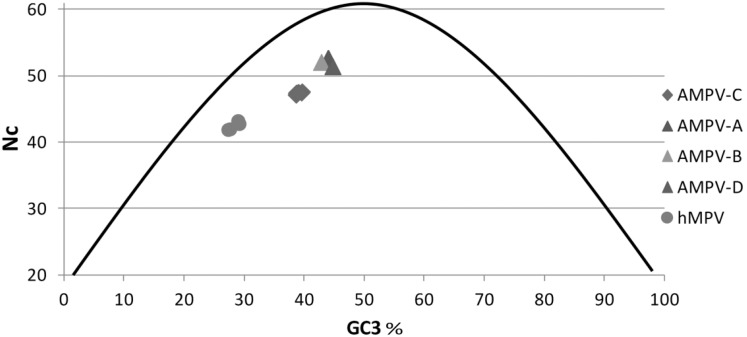
Codon usage: the effective number of codons (Nc) used within the longest MPV gene (L). Sequences included as in Table 1. The curve represents expected codon usage in relationship to the GC3 content, therefore at 50% GC content all synonymous codons should be used equally in the absence of other factors. Nc values range from 20 when only one of the possible synonymous codons is used for each amino acid, to 61 when all synonymous codons are used. The closer the Nc value is to 20, the stronger the codon usage bias is, and the higher the degree to which codons are used non-randomly. HMPVs (Nc = 41.9 to 43.2) AMPV-Cs (Nc = 47.1 to 47.5) and AMPV-A, B, D (Nc = 51.4 to 52.7).

Different groups were revealed by the codon bias analysis i) HMPVs (Nc = 41.9 to 43.2) ii) AMPV-Cs (47.1 and 47.5) and iii) AMPV-A, B, D (51.4 to 52.7) (Fig. 6). Data points were close to the curve (expected value of Nc if the bias was solely due to the G+C content at the third position) demonstrating that the biases were mostly due to the GC content. Interestingly, AMPV-C and HMPV demonstrated a different codon bias profile, although many of their proteins shared high aa similarity, a feature that most probably reflects their adaptation to a specific host. Another striking aspect of the codon bias study was that AMPV-A, B and D had a very similar codon bias, although the genetic distances were important between these viruses (Fig. 1). It is not known whether the bias picture would change if all protein sequences in the full length genome were used, however, this has been performed for HMPV and resulted in a very similar bias [43].

### Non coding regions, intergenic regions and leader and trailer sequences

The numbers of nt between two consecutive ORFs (thus a sequence encompassing the 3′NCR of previous gene, intergenic region and 5′NCR of subsequent gene) in Fr-AMPV-C were consistent with other subgroup Cs (Table 1). The smallest (21 nt) was between M2-2/SH and the largest (187 nt) between SH/G. The only notable differences in lengths occurred between G/L of AMPV-C strains 2a/97, PL1 and PL-2 (Table 1), where these three strains exhibited 28 nts, 16 nts less than all other AMPV-Cs. The numbers of nt between consecutive ORFs in subgroups A, B and D were more similar than they were to subgroup Cs or HMPVs (Table 1). Although the typical AMPV gene start signal GGGACAAGT and gene stop signal AGTTA(Xn)Poly A [13], [90]–[92] were mostly conserved amongst AMPV subgroups, some differences were observed (Table 2).

**Table 2:** AMPV transcription start and stop sequences.

gene	AMPVsubgroup	transcription startsequence	transcription stopsequence
N		**GGGACAAGT**	**AGTTA(Xn)Poly A**
	A	………	…A.(Xn)Poly A
	B	………	…A.(Xn)Poly A
	C	………	…A.(Xn)Poly A
	D	………	…A.(Xn)Poly A
P	A	………	….(Xn)Poly A
	B	………	….(Xn)Poly A
	C	………	…C.(Xn)Poly A
	D	………	…C.(Xn)Poly A
M	A	………	…C.(Xn)Poly A
	B	………	….T(Xn)Poly A
	C	………	G….(Xn)Poly A
	D	………	….(Xn)Poly A
F	A	………	….(Xn)Poly A
	B	…G….	….(Xn)Poly A
	C	………	….(Xn)Poly A
	D	………	….(Xn)Poly A
M2	A	………	….(Xn)Poly A
	B	………	….(Xn)Poly A
	C	………	….(Xn)Poly A
	D	………	….(Xn)Poly A
SH	A	………	….(Xn)Poly A
	B	…G….	….(Xn)Poly A
	C	…G….	….(Xn)Poly A
	D	………	….(Xn)Poly A
G	A	………	….(Xn)Poly A
	B	………	…C.(Xn)Poly A
	C	………	….(Xn)Poly A
	D	………	….(Xn)Poly A
L	A	A….C.A.	….(Xn)Poly A
	B	G….C.A.	….(Xn)Poly A
	C	A….C.AG	….(Xn)Poly A
	D	….C…	….(Xn)Poly A

Sequences in bold represent consensus sequence.

We have previously described the 3′ and 5′ sequence extremities of Fr-AMPV-C [34] discussing. Here complete leader and trailer sequences showed varying levels of conservation amongst all MPV’s (67–97.5%). The highest level of conservation was seen between the leader and trailer sequences of subgroup C viruses and HMPVs (79–85%). This was consistent with the heterologous rescue of AMPV-C and HMPV minigenomes using different polymerase complexes [49], [72]. Remarkably, subgroup D had a leader sequence of 62 nt which was 7 nt longer than any MPV leader sequence reported to date.

## Conclusion

This study provides the full length genome sequences for two new AMPV strains including the first full length sequence for AMPV subgroup D. Results supported previous reports that AMPV-C viruses are indeed more closely related with HMPVs than they are with other AMPV subgroups, and further demonstrate that AMPV-D is more closely related with the AMPV-A and B subgroups. Ideally, this study might be extended by sequencing more AMPV-D isolates. Unfortunately only two such isolates are currently available worldwide, both isolated in France, on the same date and within close proximity, consequently efforts to obtain new AMPV-D isolates should be continued. The three MPV “clusters” HMPV, AMPV-C and AMPV-ABD were also further supported based on phylogenetics, sequence comparisons and codon bias studies.

These data combined with those of previous reports indicating antigenic relationships between subgroups A, B and D [20], [70] and between subgroup C and HMPV [93] may call for a sub classification of MPVs comparable to that implemented for avian paramyxovirus, where viruses are first grouped into serotypes (type number) then separated into genotypes [94], [95]. Transposing a similar approach into the MPV genus would result in grouping AMPV-A, B and D as type I MPVs and AMPV-Cs and HPMVs as type II.

## Supporting Information

Table S1 **Partial nucleotide sequences previously released for Fr-AMPV-C and Fr-AMPV-D.**
(DOCX)
