# Supplementary material for: Molecular Comparisons of Full Length Metapneumovirus (MPV) Genomes, Including Newly Determined French AMPV-C and –D Isolates, Further Supports Possible Subclassification within the MPV Genus
Source: PLoS One. 2014 Jul 18;9(7):e102740. doi: 10.1371/journal.pone.0102740 (PMC4103871; doi:10.1371/journal.pone.0102740)
Supplement: Table S1 — Partial nucleotide sequences previously released for Fr-AMPV-C and Fr-AMPV-D. (DOCX) [file pone.0102740.s001.docx]

**Suppl Table S1 :** Partial nucleotide sequences previously released for Fr-AMPV-C and Fr-AMPV-D

|  | **N**  *Acc No*  (isolate) [length] | **P** | **F** | **G** | **L** | Extremities | Full genome size nt  (as per present study) | % previously known |
| --- | --- | --- | --- | --- | --- | --- | --- | --- |
| **Fr-AMPV C** | *AM293284* (99350)[1185] | *AM411376* (99350) [885] |  | *AJ811993,1* (99178) [1771] |  | 3' end [21] 5' end [20]^1^ | 14152 | 27 |
|  |  |  |  | *AJ811992,1* (99350) [1771] |  |  |  |  |
|  |  |  |  | *AJ811991,1* (00094) [1771] |  |  |  |  |
| **Fr-AMPV D** | *AM980996,1* (85035) [1176] |  | *AJ400730,1* (Fr/85/2) [partial, 323] | *AJ288946,1* (85053) [1185] | *AJ400731,1* (85053) [306] |  | 13415 | 22 |
|  |  |  | *AJ400728,1* (Fr/85/1) [partial, 323] | *AJ251085,1* (85035) [1185] | *AJ400729,1* (85035) [306] |  |  |  |

^1^ : As per Brown et Al , 2013 [35]
